# Maternal Smoking Status and Residual Risk: Current and Former Smoking Amplify Congenital Heart Disease Incidence and Severity in Offspring

**DOI:** 10.21203/rs.3.rs-9255351/v1

**Published:** 2026-04-01

**Authors:** Morgan Shaffer, Gabrielle Gray, Jennifer R. Maldonado, Jennifer A. Gaddy, Sepiso K. Masenga, Sabrina M Scroggins, Kit Neikirk, Mohd Mabood Khan, Andrea G. Marshall, Edgar Garza Lopez, Mark Santillan, Donna A Santillan, Vineeta Sharma, Linder H. Wendt, Prasanna Katti, Victoria R. Stephens, Antentor Hinton, Taneisha Gillyard, Vernat Exil

**Affiliations:** University of IOWA; University of IOWA; University of IOWA; Vanderbilt University Medical Center; Livingstone Center for Prevention and Translational Science; University of IOWA; Vanderbilt University; Vanderbilt University Medical Center; Vanderbilt University; Meharry Medical College; University of IOWA; University of IOWA; Meharry Medical College; University of IOWA; Indian Institute of Science Education and Research; Vanderbilt University Medical Center; Vanderbilt University; Meharry Medical College; University of IOWA

**Keywords:** Congenital Heart Defects, Maternal Smoking, Pregnancy Complications, Prenatal Exposure Delayed Effects, Neonatal Outcomes, Cardiovascular Abnormalities, Smoking Cessation, Fetal Development, Perinatal Risk Assessment, Tobacco Use Disorder in Pregnancy

## Abstract

**Background:**

Congenital heart disease (CHD) remains a leading cause of infant morbidity and mortality globally, and maternal smoking during pregnancy is recognized as a significant, modifiable risk factor. Despite public health efforts, a substantial proportion of women continue smoking into pregnancy, potentially exacerbating CHD risk and severity in offspring. The specific effects of smoking cessation timing on CHD outcomes remain unclear, and the residual risk associated with former smoking status requires further characterization.

**Objective(s):**

This study aimed to determine how maternal smoking status (current, former, never) influences the incidence and severity of congenital heart disease in infants, as well as associated neonatal health outcomes. The hypothesis tested was that both current and former maternal smoking increase CHD risk and severity compared to never smokers.

**Study Design:**

A cross-sectional analysis was conducted using data from 746 mother-child dyads enrolled in the University of Iowa Perinatal Family Tissue Bank from July 2010 to June 2020. Subjects included 270 CHD cases (infants diagnosed with CHD) and 476 controls without CHD. Maternal smoking status was categorized as current, former, or never, based on self-reported data at prenatal visits. CHD severity was classified as simple, moderate, or severe based on standardized clinical criteria. Statistical analyses included multivariable logistic regression to evaluate CHD incidence and severity, negative binomial regression for neonatal hospital outcomes, and Fisher’s exact tests for categorical comparisons, with significance defined as p < 0.05.

**Results:**

Current smokers exhibited significantly higher odds of fetal CHD (OR: 3.58, 95% CI: 1.95–6.68; p<0.001) compared to never smokers. Former smokers demonstrated intermediate but nonsignificant elevated risk (OR: 1.29, 95% CI: 0.82–2.00; p=0.27). The severity of CHD was greatest among current smokers (OR: 2.82; 95% CI: 1.69–4.68; p<0.001) and showed a nonsignificant upward trend among former smokers (OR: 1.45, 95% CI: 0.95–2.19; p=0.08). Infants born to current smokers required significantly longer hospitalizations (median: 13 days vs. 4 days; p=0.012) and experienced higher mortality rates at 1 year (8.2% [5/61] vs. 1.9% [10/532]; p=0.021).

**Conclusion(s):**

Maternal smoking during pregnancy significantly increases the incidence and severity of congenital heart disease in offspring. Although cessation before pregnancy reduces CHD risk, former smokers still retain residual elevated risk compared to never smokers. These findings emphasize the importance of robust public health strategies aimed at smoking prevention, cessation, and preconception counseling to mitigate the adverse impacts of maternal smoking on neonatal cardiac health.

## Tweetable Statement

Maternal smoking significantly elevates congenital heart disease risk and severity in offspring, underscoring urgent public health interventions.

## Background

Congenital heart disease (CHD) affects approximately 0.8% to 1.0% of live births globally and accounts for one-third of all major congenital anomalies, positioning it as a leading contributor to infant mortality and long-term morbidity^1–3^. Affected infants often require frequent surgical intervention, prolonged hospitalizations, and experience lower survival rates^4,5^. Despite advances in prenatal care and surgical interventions, CHD continues to impose significant psychosocial and economic burdens on families and healthcare systems^4^.

Maternal smoking during pregnancy is a well-documented modifiable factor for CHD, yet 8.5% of women in the United States report smoking during early pregnancy^6^. Prior studies have established a link between prenatal smoking and CHD incidence, but critical gaps remain in understanding the impact of cessation timing on CHD severity and perinatal outcomes^7^. Furthermore, the persistence of risk among former smokers, women who quit before pregnancy, remains poorly characterized. This study leverages a clinically annotated biorepository to address these gaps, examining the dose-dependent relationship between maternal smoking status (current, former, never) and CHD severity while evaluating associated neonatal health outcomes.

## Methods

### Study Design and Population

This cross-sectional study utilized data from participants who provided informed consent to participate in the University of Iowa Perinatal Family Tissue Bank (PFTB), a biorepository of biological samples and clinical data collected from pregnant women between July 2010 and June 2020^8^. The study population comprised 746 mother-child dyads, including 270 cases (mothers and/or infants with CHD) and 476 controls (dyads without CHD). Cases were further stratified into four groups: (1) mothers with CHD and infants without CHD (n = 15), (2) mothers without CHD and infants with CHD (n = 243), (3) mothers and infants with CHD (n = 12), and (4) controls without CHD (n = 476).

### Data Source and Ethical Approval

De-identified clinical data and biological samples were obtained from the PFTB under Institutional Review Board approval (IRB# 200910784). Longitudinal data were collected from prenatal visits, delivery outcomes, and pediatric follow-up encounters. Maternal smoking status, medical history, and infant outcomes were extracted from electronic health records (EHRs) and self-reported questionnaires.

### Inclusion and Exclusion Criteria

Women with preexisting diabetes (type I or II) or gestational diabetes were excluded due to the established association between maternal diabetes and CHD risk. To maintain a 2:1 control-to-case ratio, dyads were screened for CHD using ICD-10 codes. Infants with chromosomal abnormalities or extracardiac malformations were excluded to minimize confounding.

### Definitions of Maternal Smoking Status

Maternal smoking status was self-reported at the first prenatal visit and categorized as follows:

**Current smokers:** Women who smoked through the weeks of pregnancy, an essential window for fetal cardiac development.
**Former smokers:** Women who reported a history of smoking but had quit prior to their first prenatal visit.
**Never smokers:** Women with no lifetime history of smoking.

### CHD Classification and Severity

CHD diagnoses were validated by pediatric cardiologists using echocardiography, surgical reports, and autopsy records. Severity was classified as simple, moderate, or severe based on Task Force 1 criteria from the 32nd Bethesda Conference, which emphasizes anatomical complexity rather than intervention requirements.

For example:

**Simple CHD** included isolated atrial septal defects
**Severe CHD** included single-ventricle physiology or cyanotic lesion

### Statistical Analysis

Univariate comparisons of demographic and clinical variables across smoking groups were conducted using Fisher’s exact test for categorical variables and the Kruskal–Wallis test for continuous variables. Multivariable logistic regression models assessed the association between maternal smoking status and CHD incidence, adjusting for maternal age, gestational age at delivery, and family history of CHD. Cumulative logistic regression evaluated predictors of CHD severity (categorized as none, simple, moderate, or severe). Neonatal outcomes, including length of hospital stay and respiratory support duration, were analyzed using negative binomial regression to account for overdispersion. All analyses were performed using R version 4.1.2 and SAS 9.4, and statistical significance was defined as p < 0.05 throughout the manuscript due to the exploratory nature of the analysis.

### Ethical Considerations

The study adhered to ethical guidelines for retrospective research involving coded human data. Informed consent was obtained during enrollment in the PFTB, and the University of Iowa IRB granted a waiver for additional consent due to the use of anonymized records.

## Results

### Association Between Maternal Smoking and CHD Incidence

**Figure 1:**
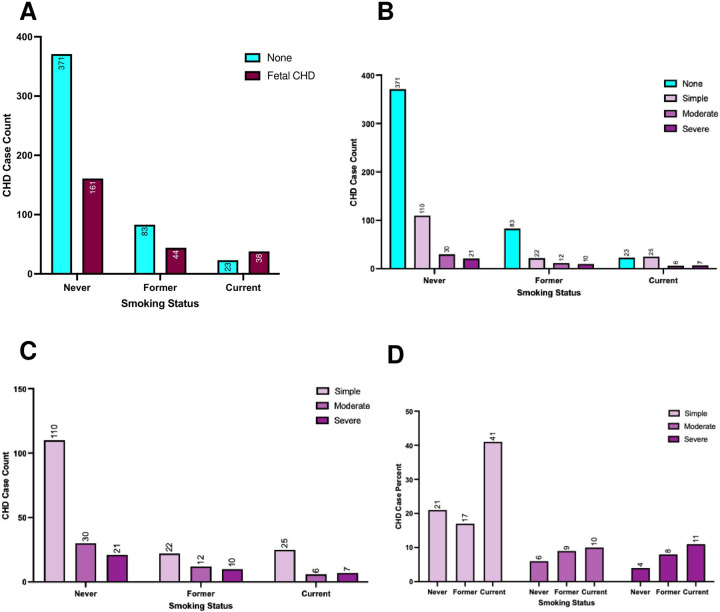
Smoking as a Risk Factor for Fetal CHD (A) Relative fetal CHD case comparison on basis of smoking status (n = 720), with never (n=532) representing self-reporting having never smoked, former (n=127) representing individuals who quit before their first obstetric appointment, and current (n=61) representing individuals who are currently smoking (p<0.001). (B) Relative complexity of fetal CHD on the basis of smoker status (p<0.001). (C) When removing those who report no CHD, changes in both count and (D) rate of individuals with simple, moderate, or severe CHD complexity may better be analyzed.

This study revealed a definite association linking maternal smoking status and both the incidence of congenital heart disease (CHD) in offspring and the severity of neonatal outcomes. Maternal smoking status was a meaningful predictor of CHD occurrence (p<0.001; Figure 1A), with current smokers exhibiting the highest proportion of CHD cases (62.3% of current smokers’ infants had CHD vs. 30.3% among never smokers). Subgroup analyses analyzing only CHD cases (Figure 1C, D) did not show a significant difference between the CHD severity levels of infants born to the different maternal smoking statuses. Current smokers exhibited 3.58-fold higher odds of having an infant with CHD compared to never smokers (95% CI: 1.95–6.68, p<0.001; Table 1), while former smokers showed no statistically significant difference in CHD incidence relative to never smokers (OR=1.29, p=0.27; Table 1). Infants born to current smokers were more likely to present with severe CHD (OR=2.82 vs. never smokers, p<0.001; Table 1, Figure 1B), while former smokers demonstrated a nonsignificant trend toward higher severity (OR=1.45, p=0.08; Table 1).

### Maternal smoking status as a predictor of quality-of-life outcomes

**Figure 2:**
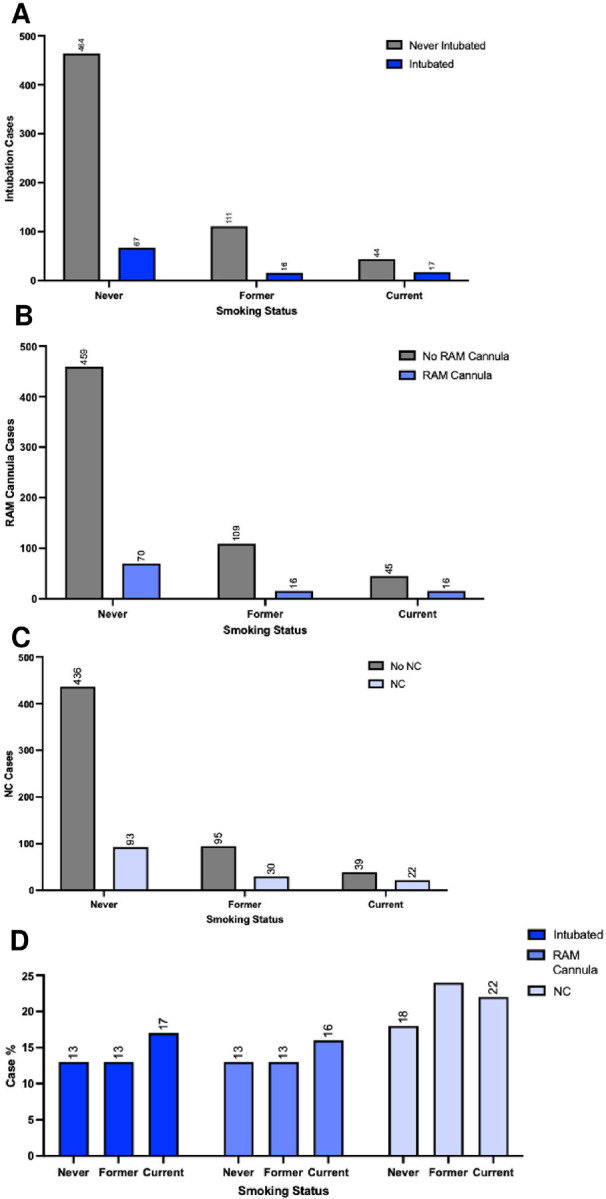
Smoking as a Risk Factor for Respiratory Support. (A) Relative intubation occurrence comparison on basis of smoking status (n = 720), with never (n=532) representing self-reporting having never smoked, former (n=127) representing individuals who quit before their first obstetric appointment, and current (n=61) representing individuals who are currently smoking (p<0.01). (B) Occurrence across smoker statuses of RAM cannula days (p<0.05), and (C) nasal cannula [NC] days (p<0.01). (D) Comparison of rates (%) of intubation, RAM cannula, and NC across individuals with varied smoker status. Significance assessed using Fisher’s exact test. N = 719 for intubated, n=715 for RAM cannula and NC given 4 missing values.

**Figure 3:**
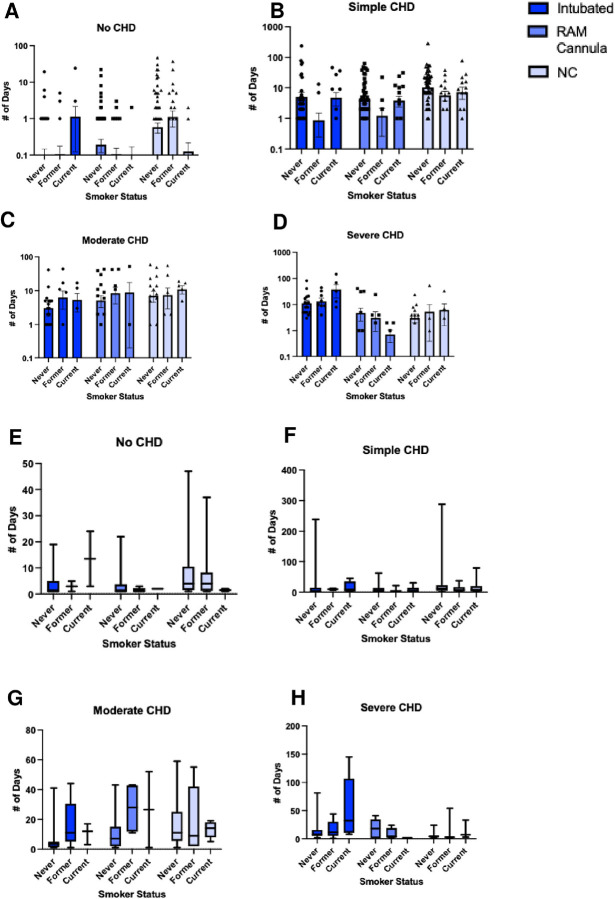
Smoking and CHD Complexity Alters Days Spent in Respiratory Support. (A) Amount of days spent in intubation, RAM cannula, and NC comparison on basis of smoking status (n = 720). (B) simple CHD, (C) moderate CHD, and (D) severe CHD. (E-H) Values of 0 days were removed, and intubation, RAM cannula, and NC days compared across smoker statues of babies with (E) no CHD, (F) simple CHD, (G) moderate CHD, and (H) severe CHD. N = 719 for intubated, n=715 for RAM cannula and NC given 4 missing values.

**Figure 4:**
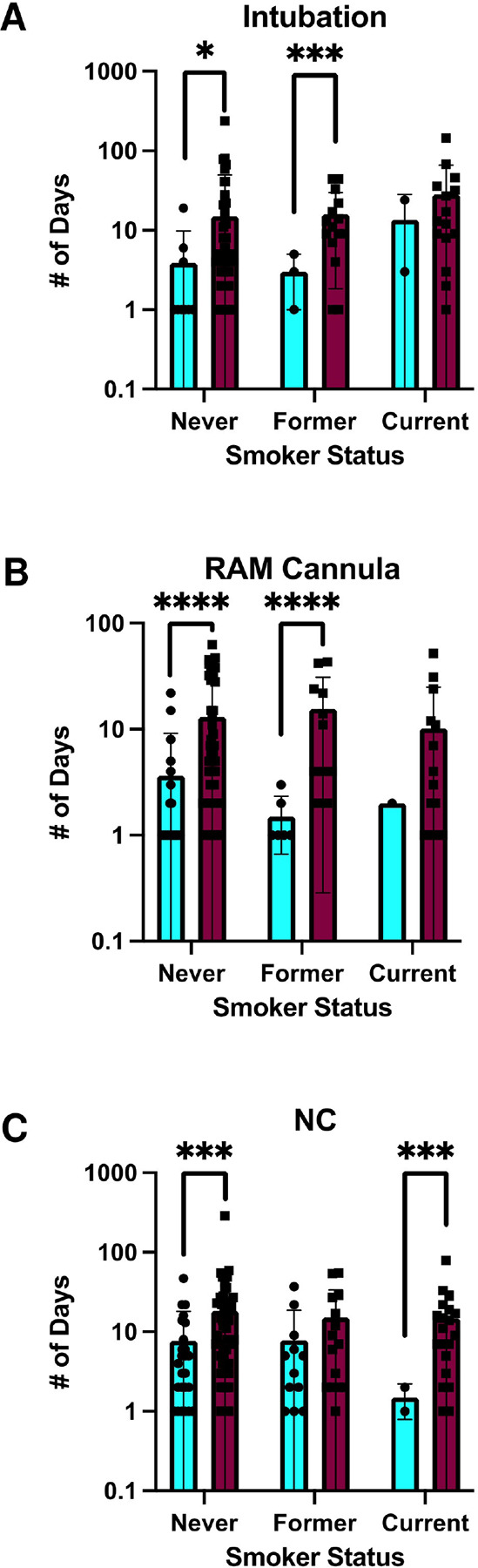
Smoking and CHD Status Alters Days Spent in Respiratory Support. (A) Values of 0 days were removed, and (A) intubation, (B) RAM cannula, and (C) NC days compared across maternal smoking statuses of babies with and without CHD. Dots represent days spent and sample number (n varies from 1–67). *** indicates p < 0.001, and **** indicates p < 0.0001.

**Figure 5:**
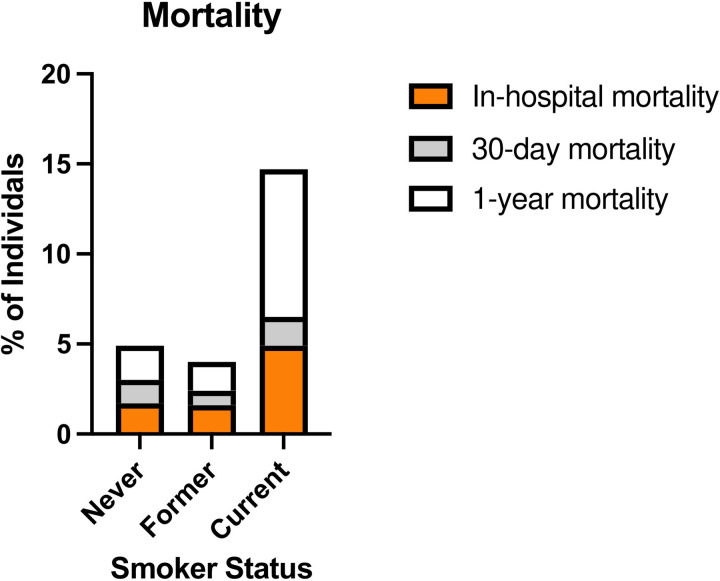
Smoker Status is Significantly Associated with Infant Mortality. All infant mortalities had previously been diagnosed with CHD. Total mortality of entire sample (n = 719) represents 40 individuals (approximately 5.6%). In-hospital (n = 14), 30-day (n=9) and 1-year (n=17) mortality considered for children of individuals with varied smoker status. Percent of individuals represents % of mortalities within the smoker status cohort. No significant differences were found between smoker status groups for in-hospital mortality (p=0.240) and 30-day mortality (p=0.725); however, there is a significant difference in 1-year mortality (p=0.021). Fisher’s exact test was performed to evaluate differences in mortality rates.

The infants’ first year of life was examined for various quality-of-life endpoints, including need for respiratory support (intubation, RAM cannula, and/or NC) (Figures 2A–D), the newborns’ length of hospital stay from birth to first discharge (Figure 3A–F and Figure 4A–C), and/or early death by 1 month and 1 year of age, according to the three maternal smoking status categories (Figure 5).

Respiratory support requirements differed markedly according to maternal smoking status. Infants of current smokers demonstrated significantly higher rates of intubation (28% vs. 13%; p = 0.003), RAM cannula use (26% vs. 13%; p = 0.011), and nasal cannula dependency (36% vs. 18%; p = 0.002) compared to infants of never smokers. Former smokers did not differ significantly from never smokers in any of these three categories. These disparities persisted across CHD severity strata. Among infants with severe CHD, offspring of current smokers required significantly longer intubation and nasal cannula support. Infants without CHD whose mothers were former smokers demonstrated greater nasal cannula dependency relative to infants without CHD whose mothers were never smokers, highlighting possible systemic effects independent of congenital anomalies. However, no other comparisons of respiratory support outcomes between former/current smokers and never smokers were significant among infants without CHD. Hospitalization outcomes underscored the clinical burden of maternal smoking. Infants of current smokers faced extended hospital stays (median: 13 days vs. 4 days for never smokers; p=0.012; Figure 4A) and elevated 1-year mortality (8.2% vs. 1.9%; p=0.021; Figure 5). Notably, 16 of 17 (94.1%) of deaths at one year occurred in CHD cases, with current smokers’ infants constituting 29.4% of 1-year mortality despite representing only 8.5% of the cohort (Figure 5). These findings collectively emphasize that maternal smoking exacerbates CHD severity and amplifies neonatal morbidity and mortality.

The analysis of risk factors associated with congenital heart disease (CHD) revealed significant differences in maternal age and gestational age across smoking groups, while family history of CHD did not reach statistical significance (Table 2). Maternal age differed significantly among groups (p= 0.033, Kruskal–Wallis test), with current smokers being younger on average (mean ± SD: 28.2 ± 6.0 years) compared to never smokers (29.9 ± 5.0) and former smokers (30.1 ± 5.5). Moreover, gestational age at delivery was significantly shorter for current smokers (259 ± 32 days) relative to never smokers (267 ± 21 days) and former smokers (266 ± 22 days) (p= 0.004).

These findings suggest that maternal smoking status is associated with younger maternal age and reduced gestational age at delivery, both of which may contribute to adverse fetal outcomes.

## Comment:

### Principal Findings

This study demonstrates that maternal smoking, both current and former, is associated with an increased incidence and severity of congenital heart disease (CHD) in offspring. Current smokers had significantly higher odds of bearing infants with CHD and more severe forms of the disease. Former smokers displayed an intermediate, though not statistically significant, increase in CHD risk and severity. Neonates born to current smokers experienced prolonged hospitalization and increased 1-year mortality. These findings establish a clear dose-dependent association between maternal smoking status and adverse neonatal cardiac outcomes.

### Results in the Context of What is Known

Previous research has established maternal smoking as a risk factor for CHD, with particular associations with septal and right-sided cardiac anomalies^4,9–11^. This study supports and extends those findings by incorporating maternal smoking status stratification (current, former, never) and demonstrating a graded risk pattern. Unlike some prior studies which focused solely on active smoking during pregnancy, our results suggest that former smokers also retain elevated risk^12–15^. This may reflect cumulative exposure or unresolved epigenetic effects. In contrast with studies that examined only incidence, we included CHD severity and neonatal outcomes, further enriching the literature^1,6,7^.

### Clinical Implications

These findings reinforce the importance of smoking prevention strategies before conception. Although cessation prior to pregnancy reduced CHD risk compared to ongoing smoking, former smokers still carried intermediate risk, indicating possible persistent biological effects. Clinicians should counsel women of reproductive age not only on the dangers of smoking during pregnancy but also on the long-term reproductive consequences of past tobacco use. Public health interventions should be broadened to include smoking cessation before conception and discourage initiation entirely.

### Research Implications

Further research is needed to delineate the biological mechanisms linking former maternal smoking with CHD risk, including the role of epigenetic modifications and vascular injury. Biochemical verification of smoking status and more precise classification of cessation timing could refine future risk stratification. Additionally, genomic and environmental interaction studies are warranted to identify vulnerable populations and inform targeted prevention.

### Strengths and Limitations

Strengths of this study include the use of a well-characterized biorepository, standardized CHD classification, and detailed neonatal outcome data. The stratification of maternal smoking status allowed for nuanced analysis of residual risks. However, reliance on self-reported smoking status introduces potential misclassification bias. The timing and duration of smoking cessation among former smokers were not captured, limiting the assessment of dose-response effects. Additionally, the sample size limited the power for CHD subtype analyses, and genetic data were not available to explore gene-environment interactions.

## Conclusions

This study underscores that both current and former maternal smoking are linked to elevated CHD incidence and severity in offspring. Smoking prevention and preconception counseling should be integral components of maternal healthcare to mitigate long-term cardiac risks in children. Broader epidemiological and mechanistic studies are necessary to confirm these findings and guide policy and clinical practice.

## Data Availability

All data is available upon reasonable request from the corresponding author

## AJOG at a Glance:

### A. Why was this study conducted?

- To evaluate how maternal smoking status (current, former, never) influences congenital heart disease (CHD) incidence, severity, and neonatal outcomes.

### B. What are the key findings?

- Current smokers had 3.58-fold higher odds of CHD in offspring.
- Former smokers exhibited intermediate risk, with a trend toward increased CHD severity.
- Maternal smoking correlated with prolonged neonatal hospitalizations and increased 1-year mortality.

### C. What does this study add to what is already known?

- It demonstrates that both current and former smoking during pregnancy amplify CHD risk and severity, highlighting persistent risk even after smoking cessation before pregnancy.
- Challenges assumptions that preconception smoking cessation fully eliminates CHD risk, highlighting the need for early prevention strategies for females.

## References

[1] Bouma BJ, Mulder BJM. Changing Landscape of Congenital Heart Disease. Circ Res.2017;120(6):908–922. doi:10.1161/CIRCRESAHA.116.309302 28302739

[2] Levis DM, Stone-Wiggins B, O’Hegarty M, Women’s perspectives on smoking and pregnancy and graphic warning labels. Am J Health Behav. 2014;38(5):755–764. doi:10.5993/AJHB.38.5.13 24933145 PMC4706759

[3] van der Linde D, Konings EEM, Slager MA, Birth prevalence of congenital heart disease worldwide: a systematic review and meta-analysis. J Am Coll Cardiol. 2011;58(21):2241–2247.doi:10.1016/j.jacc.2011.08.025 22078432

[4] Correa A, Levis DM, Tinker SC, Cragan JD. Maternal cigarette smoking and congenital heart defects. J Pediatr. 2015;166(4):801–804. doi:10.1016/j.jpeds.2015.01.013 25681204 PMC4406351

[5] Oster ME, Lee KA, Honein MA, Riehle-Colarusso T, Shin M, Correa A. Temporal trends in survival among infants with critical congenital heart defects. Pediatrics. 2013;131(5):e1502–1508. doi:10.1542/peds.2012-3435 23610203 PMC4471949

[6] Yüce B, Tengiz Fİ. Effects of tobacco use during pregnancy on infant and child health. D J Med Sci. 2020;6(2):070–073. doi:10.5606/fng.btd.2020.25024

[7] Curtin SC, Matthews TJ. Smoking Prevalence and Cessation Before and During Pregnancy: Data From the Birth Certificate, 2014. Natl Vital Stat Rep. 2016;65(1):1–14. 26905977

[8] Santillan MK, Leslie KK, Hamilton WS, Collection of a lifetime: a practical approach to developing a longitudinal collection of women’s healthcare biological samples. Eur J Obstet Gynecol Reprod Biol. 2014;179:94–99. doi:10.1016/j.ejogrb.2014.05.023 24965987 PMC4148073

[9] Ezegbe C, Neil AL, Magnussen CG, Maternal smoking during pregnancy: Trends and determinants in the conception to community study. Birth. 2021;48(1):76–85. doi:10.1111/birt.12515 33274444

[10] Alverson CJ, Strickland MJ, Gilboa SM, Correa A. Maternal smoking and congenital heart defects in the Baltimore-Washington Infant Study. Pediatrics. 2011;127(3):e647–653. doi:10.1542/peds.2010-1399 21357347

[11] Dhanantwari P, Lee E, Krishnan A, Human cardiac development in the first trimester: a high-resolution magnetic resonance imaging and episcopic fluorescence image capture atlas. Circulation. 2009;120(4):343–351. doi:10.1161/CIRCULATIONAHA.108.796698 19635979 PMC3411176

[12] Knopik VS, Maccani MA, Francazio S, McGeary JE. The Epigenetics of Maternal Cigarette Smoking During Pregnancy and Effects on Child Development. Dev Psychopathol. 2012;24(4):1377–1390. doi:10.1017/S0954579412000776 23062304 PMC3581096

[13] Zong D, Liu X, Li J, Ouyang R, Chen P. The role of cigarette smoke-induced epigenetic alterations in inflammation. Epigenetics & Chromatin. 2019;12(1):65. doi:10.1186/s13072-019-0311-8 31711545 PMC6844059

[14] Tindle HA, Stevenson Duncan M, Greevy RA, Lifetime Smoking History and Risk of Lung Cancer: Results From the Framingham Heart Study. J Natl Cancer Inst. 2018;110(11):1201–1207. doi:10.1093/jnci/djy041 29788259 PMC6235683

[15] Duncan MS, Freiberg MS, Greevy RA, Kundu S, Vasan RS, Tindle HA. Association of Smoking Cessation With Subsequent Risk of Cardiovascular Disease. JAMA. 2019;322(7):642–650. doi:10.1001/jama.2019.10298 31429895 PMC6704757

